# Identification of a Golgi-localized UDP-N-acetylglucosamine transporter in *Trypanosoma cruzi*

**DOI:** 10.1186/s12866-015-0601-7

**Published:** 2015-11-21

**Authors:** Carlos Gustavo Baptista, Elizabeth Cristina Rodrigues, Patricia Morking, Amanda Klinke, Maria Luiza Zardo, Maurílio José Soares, Alessandra Melo de Aguiar, Samuel Goldenberg, Augusto Savio Peixoto Ramos

**Affiliations:** Present address: Departamento de Parasitologia, Instituto de Ciências Biomédicas, Universidade de São Paulo, São Paulo, 05508-000 SP Brazil; Present address: Departamento de Imunologia, Instituto de Ciências Biomédicas, Universidade de São Paulo, São Paulo, 05508-900 SP Brazil; Instituto Carlos Chagas, Fiocruz Paraná, Curitiba, 81350-010 PR Brazil

**Keywords:** Glycoconjugates, Nucleotide sugar, Transporter, Trypanosomatids

## Abstract

**Background:**

Nucleotide sugar transporters (NSTs) play an essential role in translocating nucleotide sugars into the lumen of the endoplasmic reticulum and Golgi apparatus to be used as substrates in glycosylation reactions. This intracellular transport is an essential step in the biosynthesis of glycoconjugates.

**Results:**

We have identified a family of 11 putative NSTs in *Trypanosoma cruzi*, the etiological agent of Chagas’ disease. A UDP-*N*-acetylglucosamine transporter, TcNST1, was identified by a yeast complementation approach. Based on a phylogenetic analysis four candidate genes were selected and used for complementation assays in a *Kluyveromyces lactis* mutant strain. The transporter is likely expressed in all stages of the parasite life cycle and during differentiation of epimastigotes to infective metacyclics. Immunofluorescence analyses of a GFP-TcNST1 fusion protein indicate that the transporter is localized to the Golgi apparatus. As many NSTs are multisubstrate transporters, we also tested the capacity of TcNST1 to transport GDP-Man.

**Conclusions:**

We have identified a UDP-N-acetylglucosamine transporter in *T. cruzi*, which is specifically localized to the Golgi apparatus and seems to be expressed, at the mRNA level, throughout the parasite life cycle. Functional studies of TcNST1 will be important to unravel the role of NSTs and specific glycoconjugates in *T. cruzi* survival and infectivity.

**Electronic supplementary material:**

The online version of this article (doi:10.1186/s12866-015-0601-7) contains supplementary material, which is available to authorized users.

## Background

*Trypanosoma cruzi* is the etiological agent of Chagas’ disease, which affects approximately 7 million people, mostly in Latin America, but also in other regions due to migration and blood transfusion [1]. The life cycle of this parasite is complex, involving at least four well-defined life forms: the proliferative forms present in both the insect vector (epimastigotes) and mammals (intracellular amastigotes) and the infectious non-dividing metacyclics (insect stage) and bloodstream trypomastigotes [2].

The dense glycocalyx of *T. cruzi* plays a fundamental role in survival and infectivity, and its molecular composition depends on the parasite’s life form. The glycocalyx is rich in glycoinositolphospholipids (GIPLs), either free or as protein-membrane anchors. Free GIPLs are major cell surface constituents of the insect stages of the parasite acting as modulators of the host immune system [3] and in epimastigote attachment to the midgut surface of the vector [4].

Mucins, the most abundant glycoproteins of *T. cruzi*, are highly O-glycosylated proteins tethered to the membrane via glycosylphosphatidylinositol (GPI) anchors. They are involved in protection against intestinal proteases in the insect vector [5] and with the attachment to and invasion of mammalian cells [6]. In terms of structure, it is important to note that the O-linked glycans of *T. cruzi* mucins are attached to serine (Ser) or threonine (Thr) residues by N-acetylglucosamine instead of N-acetylgalactosamine as it occurs in most mammalian mucins [7].

As in other eukaryotes, the synthesis of *T. cruzi* glycoconjugates occurs in the lumen of the endoplasmic reticulum (ER) and Golgi apparatus through the action of glycosyltransferases using nucleotide sugars as substrates. These sugar-activated donors must be transported across the ER and Golgi membranes by nucleotide sugar transporters (NSTs). This intracellular transport is essential for proper protein and lipid glycosylation.

NSTs comprise a family of structurally related and highly hydrophobic type III transmembrane proteins, which have been studied in different organisms, from yeast to human [8]. Based on a detailed membrane topology study of the mouse CMP-sialic acid transporter [9], these transporters are supposed to have 10 transmembrane (TM) domains with both amino- and carboxyl-termini facing the cytosol. Mutations in NSTs are associated with widespread defects in glycosylation leading to developmental diseases in mammals and loss or attenuated infectivity of human pathogens [10].

In parasitic protozoa, the role of NSTs has been investigated in *Leishmania* spp. [11, 12], *T. brucei* [13] and *Toxoplasma gondi* [14]. In *L. major*, knockout of the GDP-mannose (GDP-Man) transporter LPG2 resulted in mutants deficient in cell-surface phosphoglycan molecules displaying attenuated virulence in mice [15]. Recently, four NSTs from *T. brucei* (TbNST1-4) were characterized [13]. Silencing and knockout experiments of TbNST4, which transports UDP-*N*-acetylglucosamine (UDP-GlcNAc), UDP-*N*-acetylgalactosamine (UDP-GalNAc) and GDP-Man, resulted in glycosylation defects, but no impairment of infectivity was observed, likely due to redundancy in function.

In this study, we have identified a UDP-GlcNAc transporter from *T. cruzi* (named TcNST1) by yeast complementation in vivo. We show that TcNST1 is localized to the Golgi apparatus and that the gene is likely expressed during the parasite life cycle and in vitro metacyclogenesis—a process by which epimastigote forms differentiate into infective metacyclic trypomastigotes. This is the first experimentally characterized NST in *T. cruzi*, for which there is relatively little information regarding glycoconjugate biosynthesis.

## Results

### *Trypanosoma cruzi* NST candidates

We initially searched for putative nucleotide sugar transporters in the *T. cruzi* genome by performing Blastp searches in GeneDB [16] using characterized NSTs of different organisms as queries. We have identified a family of eleven putative NSTs (Table 1) showing considerable similarity (e-value < 1e-10) to known NSTs. Consistent with their putative assigned function, the genes code for multi-transmembrane (TM) proteins displaying between 7 and 10 TM domains (Table 1).

**Table 1:** Putative NSTs identified in the *T. cruzi* genome (Clone CL Brener)

Gene^a^	Number of amino acids	Identity in amino acids between alleles (%)	TM domains^b^
TcCLB.511517.150 (TcCLB.511737.70) (*TcNST1*)	313 (313)	99	9
TcCLB.511301.50 (TcCLB.511353.30)	316 (316)	98	8
TcCLB.510355.220 (TcCLB.506753.60)	322 (322)	97	9
TcCLB.506509.40 (TcCLB.506579.80)	347 (344)	95	9
TcCLB.509741.20 (TcCLB.507625.200)	355 (355)	96	10
TcCLB.506793.40 (TcCLB.510611.20)	387 (390)	90	10
TcCLB.504085.60 (TcCLB.507089.40)	412 (412)	93	8
TcCLB.508737.180 (TcCLB.509127.90)	444 (494)	96	9
TcCLB.511817.280 (TcCLB.510531.10)	541 (484)	98	9
TcCLB.511277.400	350	--	7
TcCLB.504057.120	358	--	9

^a^Eleven putative *T. cruzi* NSTs were identified in GeneDB by perfoming Blastp searches using characterized NSTs of different organisms as queries. For nine of the genes both haplotypes could be identified based on similarity and synteny. The hybrid diploid CL Brener strain is composed of haplotypes named Esmeraldo-like and non-Esmeraldo-like. Genes (and their respective number of amino acids) derived from Esmeraldo-like haplotypes are indicated in parentheses

^b^Number of transmembrane domains (for non-Esmeraldo like genes only) were predicted using the TMHMM Server 2.0 (http://www.cbs.dtu.dk/services/TMHMM/)

Due to the hybrid nature of the diploid CL Brener strain [17], both haplotypes of nine of the genes could be identified based on similarity and synteny (alleles are indicated in parentheses in Table 1). The alleles in each pair are at least 90 % identical to each other at the amino acid level and, with two exceptions, code for proteins of identical or similar size (up to three amino acids in difference). The genes TcCLB.509127.90 and TcCLB.511817.280, however, have an extended amino terminus when compared to their respective alleles. Comparison of the predicted protein products with the corresponding homologues in *T. cruzi* Sylvio ×10/1, a non-hybrid strain whose draft genome sequence was made available more recently [18], and also with the putative homologues in *T. brucei* and *Leishmania* spp., suggests that for both genes the amino terminal extension results from annotation errors (data not shown).

### *T. cruzi* UDP-GlcNAc transporter

It is not possible to determine the substrate specificity of a given NST by analysis of its amino acid sequence [8]. To identify UDP-GlcNAc transporters, we used a yeast complementation approach in which a *Kluyveromyces lactis* mutant deficient in the Golgi transport of UDP-GlcNAc was used [19]. This mutant has been used for the identification of UDP-GlcNAc transporters from canine cells [20], *Caenorhabditis elegans* [21] and *T. brucei* [13]. Complementation assays are based on the less intense binding of the GS-II lectin from *Griffonia simplicifolia*, which specifically recognizes terminal GlcNAc residues, to the mutant cell surface.

**Fig. 1:**
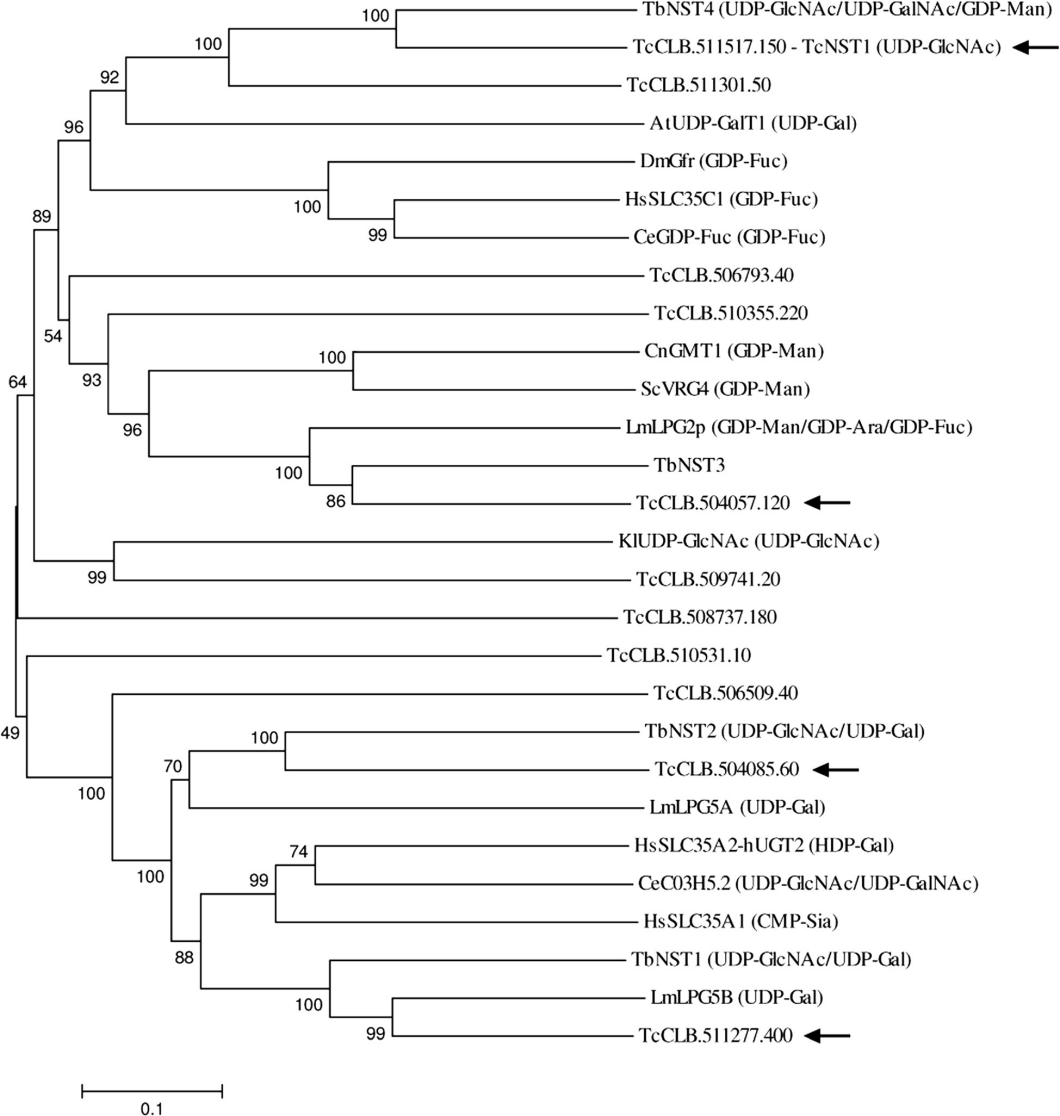
Phylogenetic analysis of NSTs from different organisms and the putative *T. cruzi* NSTs. Experimental characterized substrates are indicated in parentheses. *T. cruzi* NSTs used for complementation assays in *K. lactis* are indicated by arrows. Sequences were aligned using ClustalX2.1 and the tree was constructed with Mega5.2. Statistical method: Neighbor-Joining. Model: p-distance. Number of bootstraps replications: 1000. AtUDP-GalT1, *A. thaliana* UDP-GalT1 (21281057); CeGDP-Fuc, *C. elegans* GDP-Fuc (20138279); CeC03H5.2, *C. elegans* C03H5.2 (24636210); CnGMT1, *C. neoformans* GMT1 (68137387); DmGFR, *D. melanogaster* GFR (7299013); HsSLC35C1, *Homo sapiens* SLC35C1 (13940506); HsSLC35A2-hUGT2, *H. sapiens* SLC35A2-hUGT2 (1669566); HsSLC35A1, *H. sapiens* SLC35A1 (5453621); KlUDP-GlcNAc, *K. lactis* UDP-GlcNAc (1373152); LmPG2, *L. majo*r LPG2 (157876110); LmLPG5A, *L. major* LPG5A (45649089); LmLPG5B, *L. major* LPG5B (45649091); ScVRG4, *S. cerevisiae* VRG4 (6321213); TbNST1, *T. brucei* NST1 (Tb927.10.13900); TbNST2, *T. brucei* NST2 (Tb927.11.16560); TbNST3, *T. brucei* NST3 (Tb927.4.1640); TbNST4, *T. brucei* NST4 (Tb927.6.3960). GI numbers are indicated in parentheses

Although sequence similarity is not particularly useful for substrate specificity prediction, we used the UDP-GlcNAc transporters from *T. brucei* [13], a closely related parasite, to restrict our candidate genes. Based on phylogenetic analysis between the putative *T. cruzi* transporters and known NSTs from different organisms (Fig. 1), the genes TcCLB.511277.400, TcCLB.504085.60, TcCLB.504057.120 and TcCLB.511517.150 code for the closest transporters to TbNST1, 2, 3 and 4, respectively. These genes, indicated by arrows in Fig. 1, were then used for the complementation assays in *K. lactis*.

**Fig. 2:**
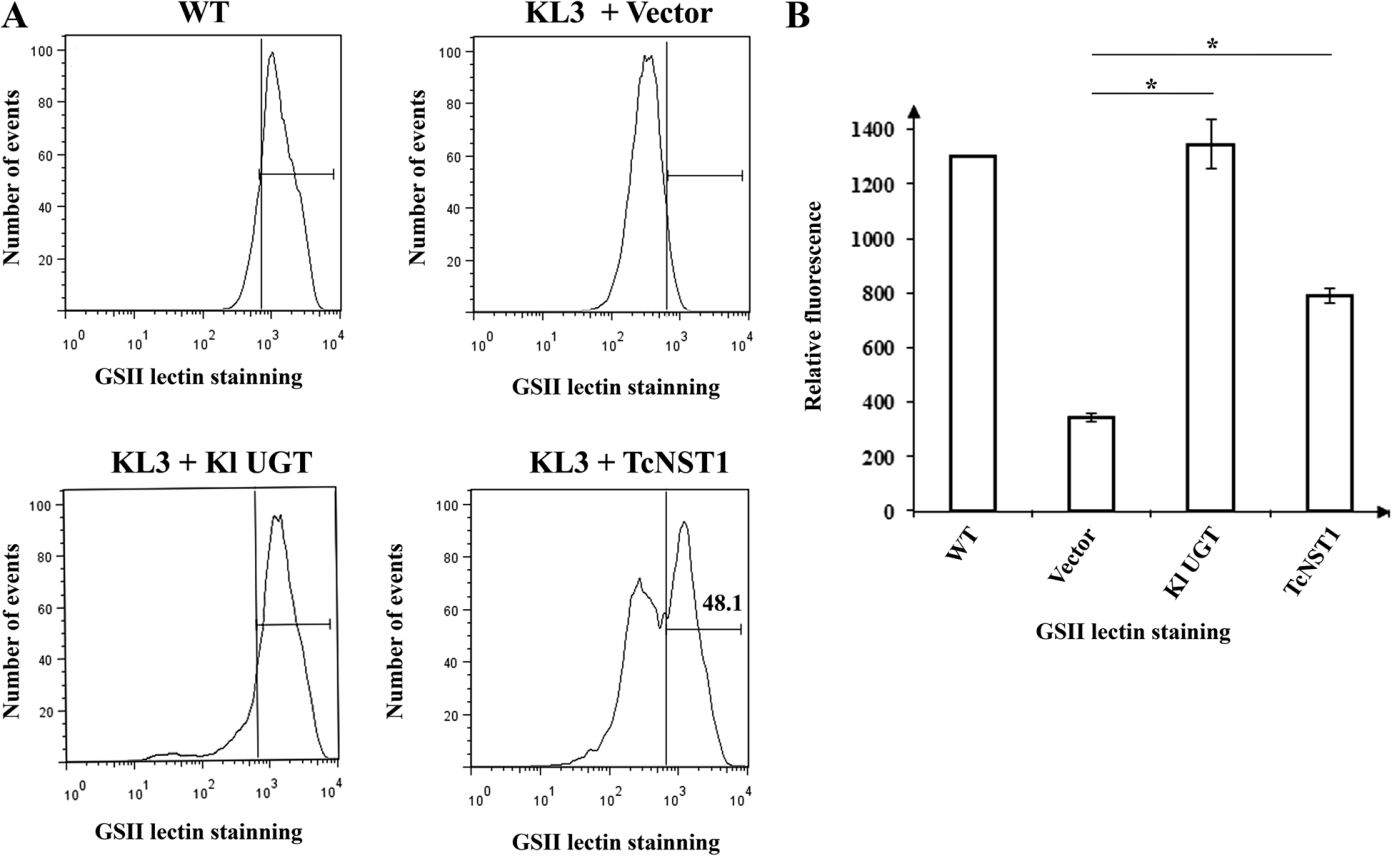
Identification of a UDP-GlcNAc transporter (TcNST1) of *T. cruzi* by in vivo complementation assays. *K. lactis* mutant (Kl3) cells were transfected with TcNST1, the *K. lactis* UDP-GlcNAc transporter (Kl UGT, positive control) or empty vector (pE4, negative control). Wild-type *K. lactis* cells were included in the analysis for comparison. Cells were grown as described in the Methods section. After labeling with GS-II lectin (Alexa Fluor 488 conjugate), yeast cells were separated by flow cytometry in a FACS Canto II (Becton & Dickinson). **a** Representative histograms showing lectin binding transfected cells. **b** Mean fluorescence intensities of a representative experiment in triplicate. Error bars represent the standard deviations. * *p* < 0.01

Only the transporter encoded by the TcCLB.511517.150 gene, TcNST1, could rescue the wild-type phenotype (Fig. 2a). Restoration of GSII lectin binding to levels similar to those of wild-type cells was observed in approximately 50 % of cells transfected with TcNST1. Similar results have been observed for the heterologous expression of NSTs in yeast, and it is likely related to the expression of nonfunctional transporters [ 22]. The mean fluorescence intensity values are presented in Fig. 2b. For cells expressing TcNST1, the fluorescence intensity corresponded to approximately 60 % of wild-type levels, a finding that is in agreement with half of the cells not restoring GS-II lectin binding. As a positive control, we used the *K. lactis* UDP-GlcNAc transporter (Kl UGT), whose activity is deficient in the yeast mutant strain. The expression of the other genes in the *K. lactis* mutant cells did not increase binding of the lectin (Additional file 1). Expression of these genes in *K. lactis* was analyzed by western blotting using a monoclonal antibody against the histidine tail inserted at the carboxyl-terminal of the transporters. However, our results regarding level of expression and apparent molecular weight were not conclusive (data not shown). Therefore, the incapacity of TcCLB60, TcCLB120 and TcCLB400 to complement the *K. lactis* mutant could be due to lack of proper expression in the yeast cells.

The TcCLB.511517.150 gene and its allele TcCLB.511737.70 are both composed of 942 nucleotides and code for a 313-amino acid polypeptide. There is only one amino acid difference between them, in which an asparagine residue is replaced by a serine (N74S) in TcCLB.511517.150. Sequencing of the gene from *T. cruzi* clone Dm28c, which was used in this work, revealed a protein identical to its homologue in *T. cruzi* Sylvio ×10/1, encoded by the gene TCSYLVIO_003299. This protein differ by three amino acids to that encoded by the gene TcCLB.511517.150 in *T. cruzi* CL Brener (K7N, L168V, and L234F in TcCLB.511517.150).

**Fig. 3:**
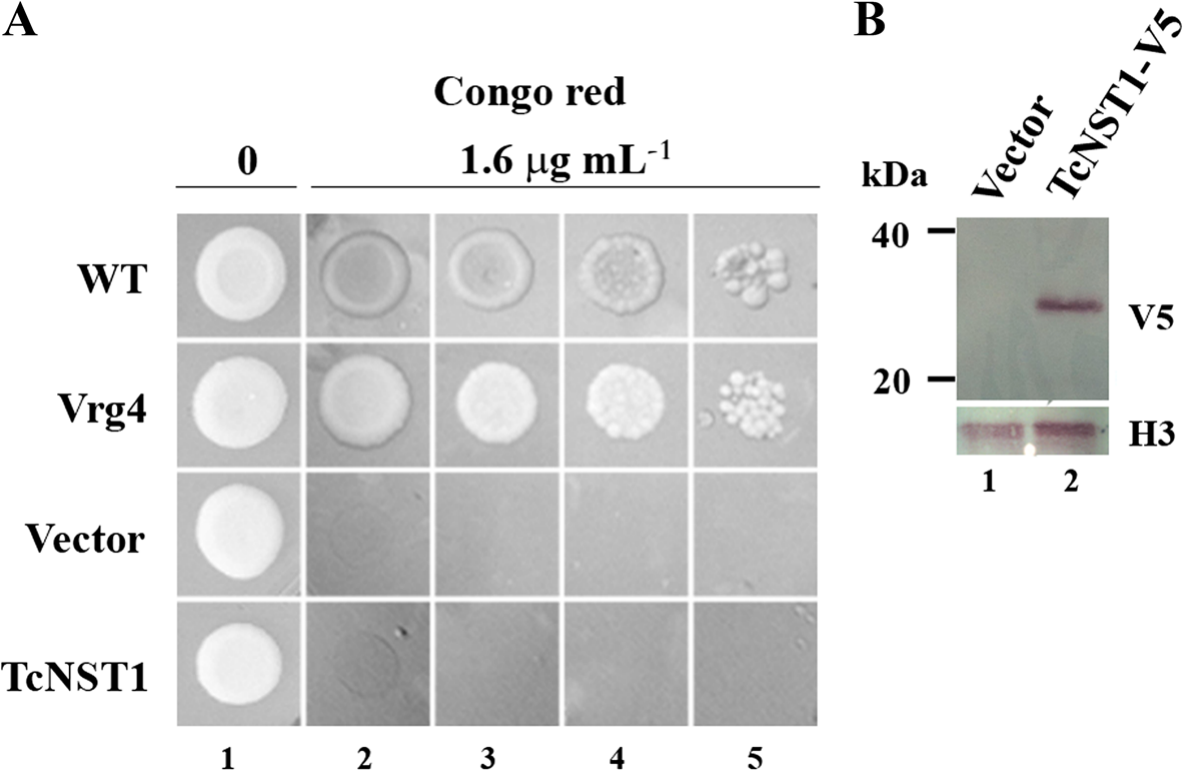
TcNST1 cannot complement a *S. cerevisiae* mutant strain deficient in GDP-Man transport. **a** *S. cerevisiae* strain NDY5 was transfected with TcNST1-V5, the *S. cerevisiae* GDP-Man Vrg4p transporter (positive control) or empty vector (pYEST-DEST 52, negative control). Wild-type *S. cerevisiae* (JPY25 6c) cells were included in the analysis for comparison. Transfected cells were grown to exponential phase in liquid cultures of SCM-URA, adjusted to an OD of approximately one (lanes 1 and 2) and submitted to ten-fold serial dilution (lanes 3–5). Cells were then inoculated on solid agar plates containing YP plus 2 % galactose and 1.6 μg/ml Congo red. Growth was assessed after 4 days at 30 °C. **b** Western blot analysis of strain NDY5 transfected with empty vector (lane 1) or TcNST1-V5 (lane 2). TcNST1 expression was detected with a mouse anti-V5 antibody. Expression of histone 3 was used as an internal control

Many NSTs are multisubstrate transporters, including the recently characterized *T. brucei* transporters. TbNST4 is the closest *T. brucei* transporter to TcNST1, and it can transport UDP-GlcNAc, UDP-GalNAc and GDP-Man. Next, we decided to test whether TcNST1 would also be able to transport GDP-Man because mannose residues are important for *T. cruzi* glycoconjugates (GalNAc residues are not present in either *T. cruzi* or *T. brucei* glycoconjugates). The capacity to transport GDP-Man was assessed by complementation of a *Saccharomyces cerevisiae* mutant partially deficient in GDP-Man transport (NDY5 strain) [23] using a fusion protein TcNST1-V5. As shown in Fig. 3a, TcNST1 could not restore growth of the yeast cells in the presence of the anionic dye Congo red. To rule out the possibility that TcNST1 was not expressed in the yeast cells, western blot analyses were carried out using an anti-V5 monoclonal antibody. TcNST1 is visualized in total protein extracts of cells transfected with the vector encoding TcNST1-V5 but not in cells transfected with the empty vector (Fig. 3b).

### TcNST1 subcellular localization and gene expression

**Fig. 4:**
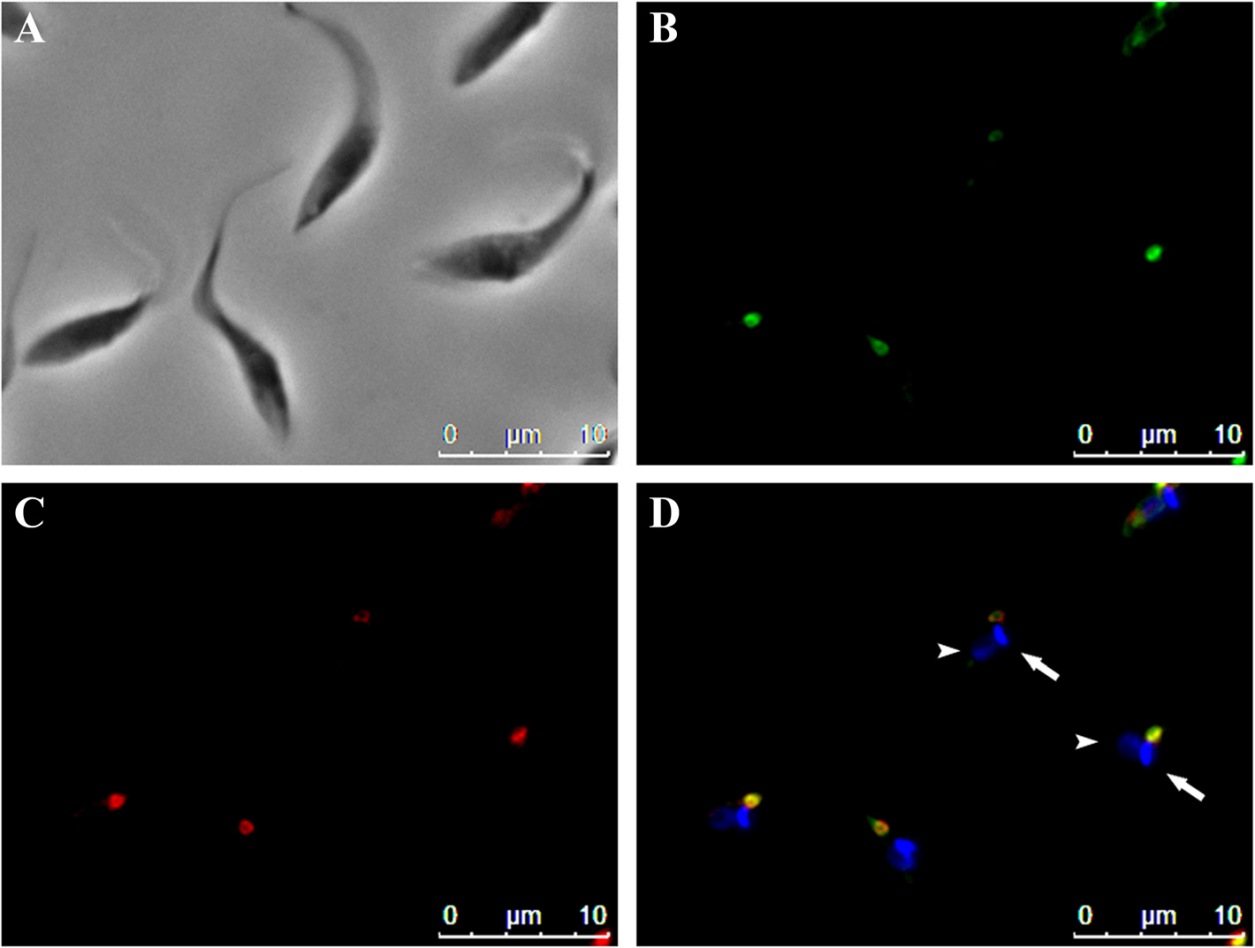
TcNST1 is localized to the Golgi apparatus. *T. cruzi* epimastigotes were transfected with a fusion construct in which TcNST1 was tagged at the amino terminus with GFP (GFP-TcNST1). Tagged TcNST1 was localized by fluorescence microscopy after staining with anti-GFP (4b, *green*) and anti-TcHIP, a Golgi marker (4c, *red*). Kinetoplasts (*large arrow*) and nuclei (*arrowhead*) were stained with DAPI (4d, *blue*). Secondary antibody controls showed no fluorescence signal (data not shown)

That TcNST1 could restore UDP-GlcNAc transport in the *K. lactis* mutant suggested the Golgi apparatus as its probable subcellular localization. To investigate this hypothesis in *T. cruzi*, we used a chimeric protein in which green fluorescent protein (GFP) was fused to the N-terminus of TcNST1. After transfection and selection of neomycin-resistant cells, *T. cruzi* epimastigotes were analyzed by fluorescence microscope. Transfected epimastigotes with the GFP-TcNST1 fusion protein displayed a dot signal at the anterior region of the parasite while fluorescence emitted by transfectants expressing only GFP-Flag (GFP fused at the C-terminus with Flag) was distributed through the cytoplasm. Wild-type cells had little intrinsic fluorescence (Additional file 2). Next we used a monoclonal antibody against GFP to detect expression of GFP-TcNST1 in total extracts of epimastigotes. As shown in Additional file 3, the transporter fused to GFP is present in transfected epimastigotes but not in wild-type cells. The Golgi localization of TcNST1 was confirmed by co-localization with TcHIP, a putative *T. cruzi* zDHHC palmitoyl transferase recently shown to be localized to the Golgi apparatus [24]. A clear punctual signal in close contact with the kinetoplast, as would be expected for a Golgi resident protein (Fig. 4b, d), was shown to co-localize with TcHIP (Fig. 4c, d).

**Fig. 5:**
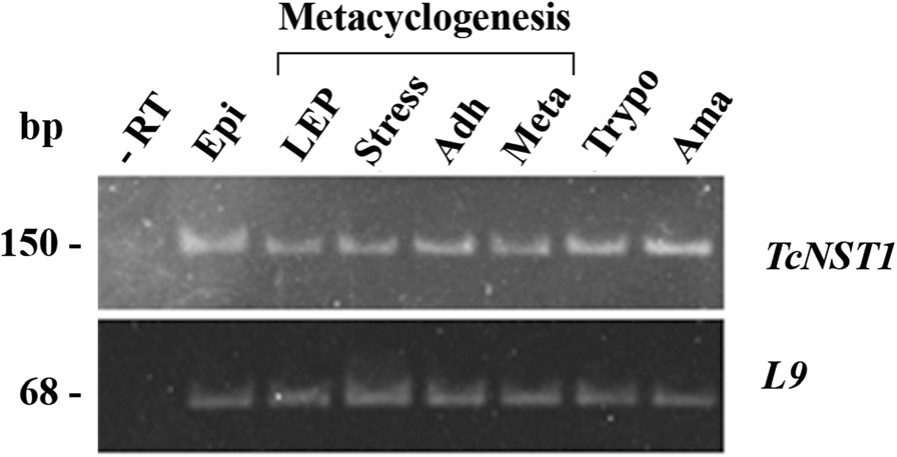
TcNST1 is expressed at the mRNA level in all life forms of *T. cruzi* and during metacyclogenesis in vitro. RT-PCR analysis of *TcNST1* mRNA expression in epimastigotes (Epi), cell-culture-derived trypomastigotes (Trypo), amastigotes (Ama), metacyclics (Meta) and intermediary forms during metacyclogenesis: epimastigotes in the late exponential phase (LEP), epimastigotes under nutritional stress (Stress) and adhered epimastigotes (Adh). Expression of the 60S ribosomal protein L9 was used as an internal control (TcCLB.504181.10). Aliquots of all samples were pooled together and subjected to reverse transcription without reverse transcriptase (− RT)

TcNST1 expression was analyzed by semi-quantitative RT-PCR along the life cycle of *T. cruzi* and during the process of metacyclogenesis in vitro, in which epimastigotes in the late exponential phase are differentiated into metacyclics under nutritional stress in a chemically defined differentiation medium [25]. In this medium, called TAU3AAG, epimastigotes adhere to a substrate and are released as metacyclics after approximately 100 h. According to the results shown in Fig. 5, *TcNST1* mRNA is present in all four major life forms of *T. cruzi* and in intermediate forms during differentiation from epimastigotes to metacyclics. While post-transcriptional control of gene expression is particularly important in trypanosomatids, the presence of mRNA suggests that the transporter may be expressed in all life forms of the parasite.

## Discussion

We have identified a family of eleven putative NSTs from *T. cruzi* (Table 1). These putative transporters show an overall structural similarity with other members of the NST family. Syntenic homologues to the TcCLB.511301.50 and TcCLB.506509.40 genes are present in *Leishmania* spp. (*L. donovani*, *L. infantum*, *L. major*, *L. mexicana*) but not in *T. brucei*. For all the other putative *T. cruzi* NSTs, there is a clear syntenic homologue in *T. brucei*. As has been noted by Capul et al. [12], an interesting role for transporters shared by *Leishmania* spp and *T. cruzi* involves UDP-galactofuranose (UDP-Galf), which is present in both trypanosomatids but not in *T. brucei*. According to our phylogenetic analyses and the TriTrypDB, two *T. cruzi* NSTs, TcCLB.510355.220 and TcCLB.506793.40, have no clear homologues in *T. brucei* or *Leishmania*. Similarly, putative roles for these transporters could be the transport of UDP-xylose (UDP-Xyl) and UDP-rhamnopyranose (UDP-Rha), which are synthesized only by *T. cruzi* [26].

Eight nucleotide sugars have been identified in *T. cruzi* by spectrometry [26], GDP-Man, UDP-GlcNAc, UDP-glucose (UDP-Glc), UDP-Gal, UDP-Galf, UDP-Xyl, UDP-Rha and GDP-fucose. Accordingly, the corresponding sugars have been identified in *T. cruzi* glycoconjugates [27]. Because we have identified eleven putative NSTs, and assuming that they are all functional, redundancy likely exists between these transporters, as has been reported in other organisms. In fact, the four recently characterized *T. brucei* NSTs can transport UDP-GlcNAc, two of them (TbNST1-2) transport UDP-Gal, and the other two (TbNST3-4) transport GDP-Man.

TcNST1 showed the greatest values of similarity (using Blastp and considering Query Coverage > 90 %) to the *T. brucei* TbNST4 transporter (79 %), the *Arabidopsis thaliana* UDP-Gal transporter UDP-GalT1 (51 %) and the *Aspergillus fumigatus* UDP-galactofuranose transporter GlfB (44 %). The *K. lactis* UDP-GlcNAc transporter, whose deficient activity in the yeast mutant strain was compensated by TcNST1, is a more distantly related transporter (Fig. 1), constituting another example of the difficulty involved in determining the substrate specificity of NSTs based on primary sequence analysis.

We also tested the capacity of TcNST1 to transport GDP-Man in yeast cells. The complementation test was negative (Fig. 3). TcNST1 was shown to be expressed in the yeast cells by western blotting, but we cannot rule out that the transporter is not functional in *S. cerevisiae* by mislocalization. Therefore, even though our results suggest that GDP-Man is not a substrate for TcNST1 in the parasite it is not a definite conclusion. A conserved GALNK motif associated with binding to GDP-Man was identified in the *S. cerevisiae* VRG4 GDP-Man transporter [28]. The same motif is present in the GDP-Man transporters of *Cryptococcus neoformans* (GMT1), *T. brucei* (TbNST3) and *L. major* (LPG2). Interestingly, in TbNST4, which transports GDP-Man, and TcNST1, this motif is less conserved, but a leucine residue, which is important for VRG4 function at physiological levels [28], is present in TbNST4 (Leu267) but not TcNST1.

NSTs are mainly localized to the Golgi apparatus. However, in trypanosomatids, nucleotide sugars are supposed to be synthesized in glycosomes [29, 30]. It was important, then, to investigate the subcellular localization of TcNST1. Our results (Fig. 4) suggest a clear Golgi localization with no indication of the presence of the transporter in glycosomes.

UDP-GlcNAc is an important substrate for the three trypanosomatids discussed here because it is required for the synthesis of both the conserved core of GPI anchors and the oligosaccharide core of N-glycans [27]. In *T. brucei*, in which the synthesis of UDP-GlcNAc is essential for parasite survival, the pool of UDP-GlcNAc inside the Golgi lumen is necessary for the synthesis of *N*-acetyllactosamine [31]. In *T. cruzi*, the Golgi content of UDP-GlcNAc is important for the synthesis of O-linked glycans in mucins, which starts with the attachment of GlcNAc, from UDP-GlcNAc, to a Ser or Thr residue via an α-*N*-acetylglucosaminyltransferase [32–34]. Therefore, the transport of UDP-GlcNAc across the Golgi membrane is essential for the proper synthesis of *T. cruzi mucins*.

## Conclusions

TcNST1 is a UDP-GlcNAc transporter specifically localized to the Golgi apparatus. Due to its specificity and subcellular localization, it is tempting to speculate that TcNST1 may have a role on the synthesis of *T. cruzi* mucins. Moreover, the expression of TcNST1, at the mRNA level, in all life forms of *T. cruzi* is in agreement with the expression of mucins throughout the life cycle of the parasite. Loss-of-function studies of this transporter are underway to unravel its role in *T. cruzi* glycosylation.

## Methods

### *Trypanosome* cultures

Epimastigotes of *Trypanosoma cruzi* Dm28c clone were maintained in liver-infusion tryptose (LIT) medium containing 10 % of fetal bovine serum (FBS) at 28 °C as previously described [35]. Transfection of epimastigotes in exponential growth phase was performed by electroporation [36] and transfectants were selected in the presence of G418 (500 μg/ ml). Metacyclics were obtained by in vitro differentiation of epimastigotes under nutritional stress using chemically defined axenic conditions as described [25]. After differentiation, metacyclic trypomastigotes were purified with ion exchange chromatography on diethylaminoethyl - cellulose columns. The metacyclics obtained after purification usually contain less than 1 % of epimastigotes-like forms. Cell-derived amastigotes and trypomastigotes were obtained by infection of VERO cells with metacyclic forms. Trypomastigotes were released in the supernatant of VERO cell cultures 4 days after infection and harvested by centrifugation. Amastigotes were obtained by disruption of VERO cells 2 days after infection. According to optical microscopic analyses cross-contamination between amastigotes and trypomastigotes are typically less than 5 %.

### Yeast strains and growth conditions

The *Kluyveromyces lactis* wild-type strain MG1/2 and mutant strain KL3 [19] were kindly provided by Dr. C.B. Hirschberg and Dr. C. Bendixen. The *Saccharomyces cerevisiae* strain JPY25 6c and the mutant strain NDY5 were kindly provided by Dr. N. Dean. Yeast strains were grown at 30 °C on rich medium (YPD) or on synthetic complete medium lacking uracil (YNB supplemented with drop-out medium without uracil, SCM-URA).

### Plasmid construction

For the complementation assays in *K. lactis*, the coding sequences of the candidate genes were first amplified by PCR from *T. cruzi* genomic DNA using specific primers and then cloned into the pE4 vector [20]. The *K. lactis* UDP-GlcNAc transporter was used as a positive control. The corresponding gene of the wild-type MG1/2 strain was amplified from genomic DNA and cloned into the same vector. The primers and restriction sizes used for cloning are indicated in Additional file 4.

The plasmids used for expression in *S. cerevisiae* and *T. cruzi* were constructed using Gateway Technology (Invitrogen). TcNST1 and the *S. cerevisiae* GDP-Man transporter ScVRG4 were first cloned into the pDONR 221 vector and then transferred by recombination to the destination vectors pYES-DEST 52 (Invitrogen) and pTcGFPN [37] for expression in *S. cerevisiae* and *T. cruzi*, respectively. The pYES-DEST 52 vector contains the V5 epitope at the carboxyl-terminus for protein detection and the pTcGFPN vector contains GFP at the amino-terminus and neomycin as a selectable marker. GFP was cloned into the vector pTcGW-3xFlag-Neo, a modified vector derived from the cloning system developed by Batista et al. [37] and kindly provided by Dr. Stenio P Fragoso. All plasmids were confirmed by sequencing.

### Complementation assays

*K. lactis* cells transfected with putative *T. cruzi* NSTs cloned into the pE4 vector [20] were grown on selective medium (SCM-URA). Cell-surface labeling with GS-II lectin was performed according to [20]. Lectin binding was analysed by flow cytometry in a FACS Canto II (Becton & Dickinson). The sensitivity of the *S. cerevisiae* strain NDY5 to Congo red was assessed by growth on solid agar plates containing YP plus 2 % galactose and 1.6 μg/ml of the dye. Galactose was used as a carbon source because the pYES-DEST 52 vector contains the yeast *GAL*1 promoter. Transfected cells in exponential growth were adjusted to an OD of approximately one and then submitted to ten-fold serial dilution. Growth was assessed after 4 days at 30 °C.

### Immunofluorescence analysis

Transfected epimastigotes expressing TcNST1 in fusion with GFP were used for subcellular localization experiments. GFP intrinsic fluorescence of epimastigotes expressing GFP-TcNST1 or GFP-Flag was visualized in live parasites. For co-localization experiments with TcHIP, cells were incubated with rabbit anti-GFP (1:400) and mouse anti-TcHIP (1:100) [24], after fixation (4 % formaldehyde), permeabilization (0.1 % Triton X-100) and blocking (2 % BSA). Secondary antibodies conjugated to Alexa 488 (goat anti-rabbit IgG) and Alexa 594 (goat anti-mouse IgG) were used. Genomic and kinetoplast DNA was visualized with 4′,6-diamidino-2-phenylindole (DAPI). Slides were examined using a Leica DMI6000 B inverted microscope.

### Western blot analysis

Total protein extracts were prepared from exponential growing yeast cells on SCM-URA supplemented with 2 % galactose. Cells were disrupted by glass beads in the presence of RIPA buffer. Cell lysates from *T. cruzi* epimastigotes (5 × 10^6^ parasites) were obtained by direct lysis in Laemmli buffer. After electrophoresis on SDS-PAGE gels (13 %), proteins were electrotransferred onto Optitran BA-S85 membranes (GE Healthcare). Membrane-bound proteins were incubated either with mouse anti-V5 antibody and rabbit anti-human histone 3 or with rabbit anti-GFP and mouse anti-GAPDH. Protein detection was performed using alkaline phosphatase or the Li-COR Odyssey imaging system.

### Total RNA isolation and reverse transcription

Total RNA was isolated from trypanosome cells using the RNeasy® Mini Kit (Qiagen). Any contaminant DNA was digested with 1 U per μg of RNA with RNase-free DNase (Promega). Aliquots of 1 μg of total RNA were then reverse transcribed to first-strand cDNA using oligo (dT) primers. Amplification by PCR was performed with the primers Tc150F4 (5′GTACTTCAAGACGGCGTTGG3′) and Tc150R4 (5′GGCTGACAGTCCGTTCATCT3′) for TcCLB.511517.150 and L9F (5′CCTTCACTGCCGTTCGTTGGTTTG3′) and L9R (5′ATGCGAGAGTGCCGTGTTGATGGT3′) for the 60S ribosomal L9 gene used as an internal control.

## Additional files

Additional file 1:
***T cruzi*** **candidate genes tested for UDP-GlcNAc transport by in vivo complementation assays.** *K. lactis* mutant (Kl3) cells were transfected with TcCLB.504057.120 (TcCLB120), TcCLB.504085.60 (TcCLB60) and TcCLB.511277.400 (TcCLB400), the *K. lactis* UDP-GlcNAc transporter (Kl UGT, positive control) or empty vector (pE4, negative control). Cells were grown as described in the Methods section. After labeling with GS-II lectin (Alexa Fluor 488 conjugate), yeast cells were separated by flow cytometry in a FACS Canto II flow cytometer (Becton & Dickinson). (TIF 1867 kb) Additional file 2:
**Intrinsic fluorescence emitted by transfected parasites expressing GFP-TcNST1.** Epimastigotes expressing the transporter fused to GFP display a single dot at the anterior region of the parasite. Live parasites from cultures of epimastigotes in exponential growth were washed once with PBS, mounted onto slides and immediately visualized in Leica DMI6000 B inverted microscope. (TIF 1386 kb) Additional file 3:
**Expression of GFP-TcNST1 in** ***T. cruzi*** **epimastigotes.** Western blot analysis of *T. cruzi* parasites transfected with GFP-TcNST1 and wild-type cells. Expression of the fusion protein (arrow) was detected with a monoclonal antibody against GFP. Expression of glyceraldehyde 3-phosphate dehydrogenase (GAPDH, ~ 37 kDa) was used as an internal control. (TIF 69 kb) Additional file 4:
**Primers used for cloning of the listed genes in different expression vectors.** (DOCX 15 kb)

## Abbreviations

ER: endoplasmic reticulum
GFP: green fluorescence protein
GIPL: glycoinositolphospholipid
GPI: glycosylphosphatidylinositol
GS-II lectin: *Griffonia simplicifolia* lectin
NST: nucleotide sugar transporter
