# Supplementary figures and images for: Identification of a Golgi-localized UDP-N-acetylglucosamine transporter in Trypanosoma cruzi
Source: BMC Microbiol. 2015 Nov 21;15:269. doi: 10.1186/s12866-015-0601-7 (PMC4654811; doi:10.1186/s12866-015-0601-7)

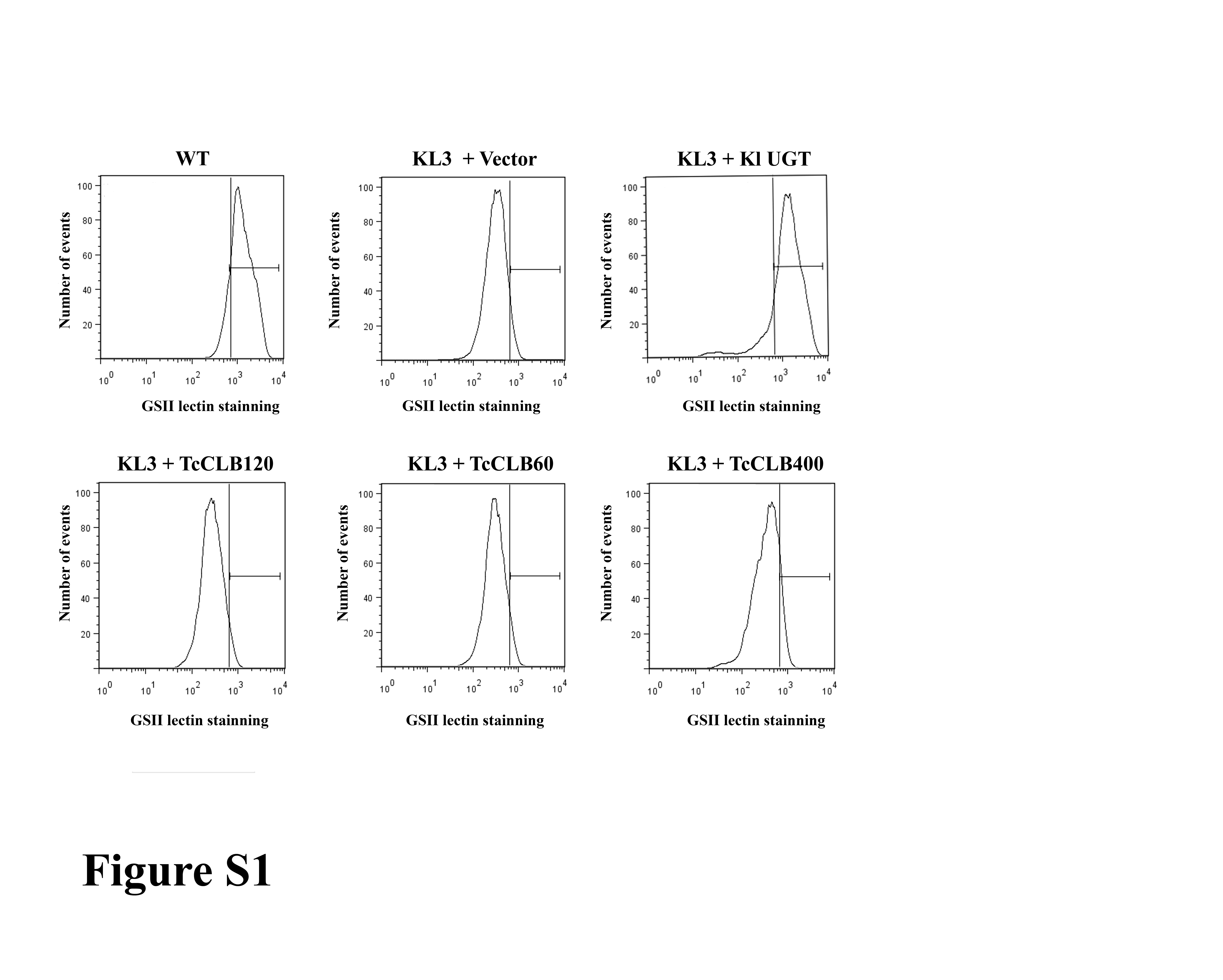

Supplement: Additional file 1: — T cruzi candidate genes tested for UDP-GlcNAc transport by in vivo complementation assays. K. lactis mutant (Kl3) cells were transfected with TcCLB.504057.120 (TcCLB120), TcCLB.504085.60 (TcCLB60) and TcCLB.511277.400 (TcCLB400), the K. lactis UDP-GlcNAc transporter (Kl UGT, positive control) or empty vector (pE4, negative control). Cells were grown as described in the Methods section. After labeling with GS-II lectin (Alexa Fluor 488 conjugate), yeast cells were separated by flow cytometry in a FACS Canto II flow cytometer (Becton & Dickinson). (TIF 1867 kb) [file 12866_2015_601_MOESM1_ESM.tif]

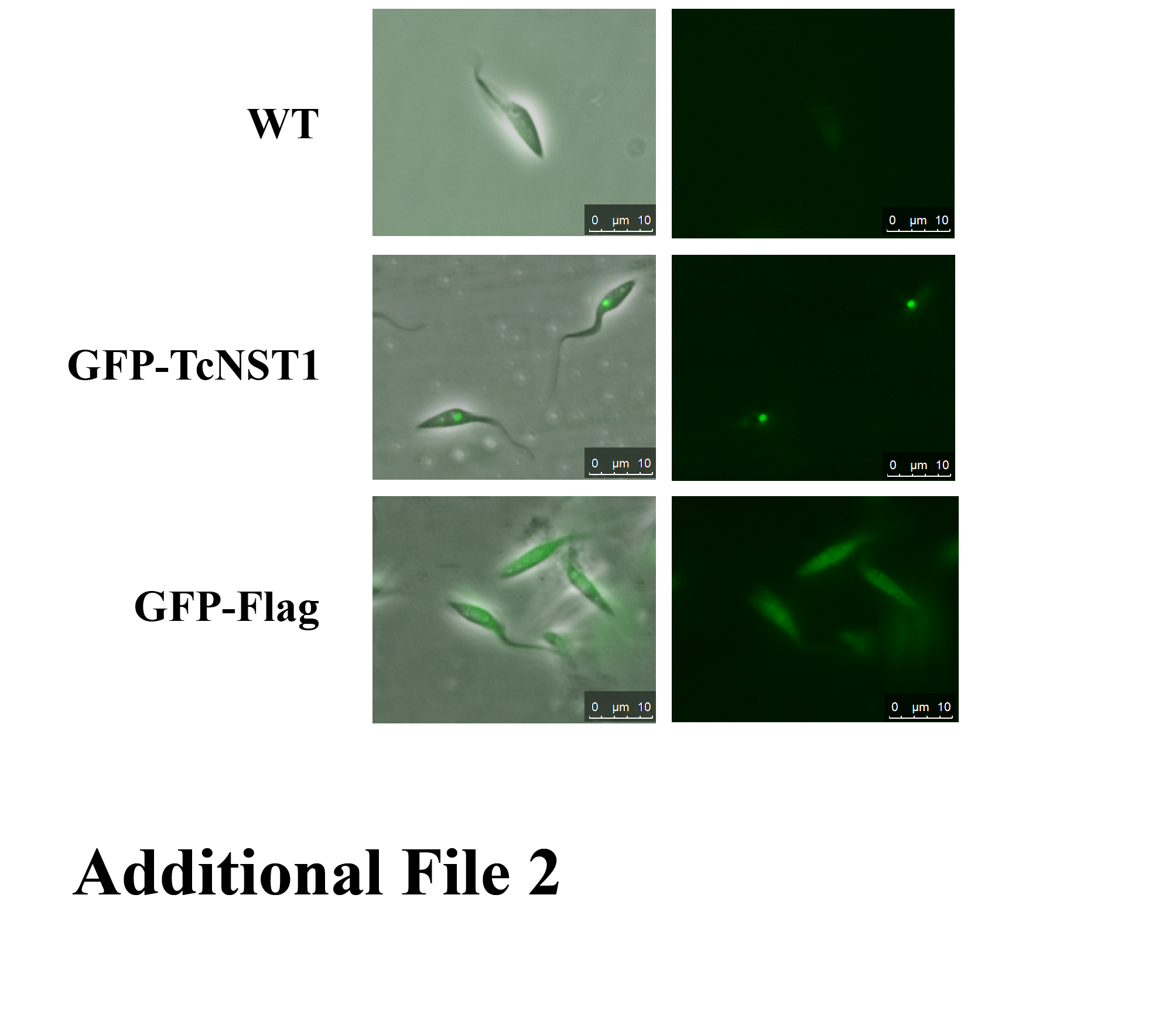

Supplement: Additional file 2: — Intrinsic fluorescence emitted by transfected parasites expressing GFP-TcNST1. Epimastigotes expressing the transporter fused to GFP display a single dot at the anterior region of the parasite. Live parasites from cultures of epimastigotes in exponential growth were washed once with PBS, mounted onto slides and immediately visualized in Leica DMI6000 B inverted microscope. (TIF 1386 kb) [file 12866_2015_601_MOESM2_ESM.tif]

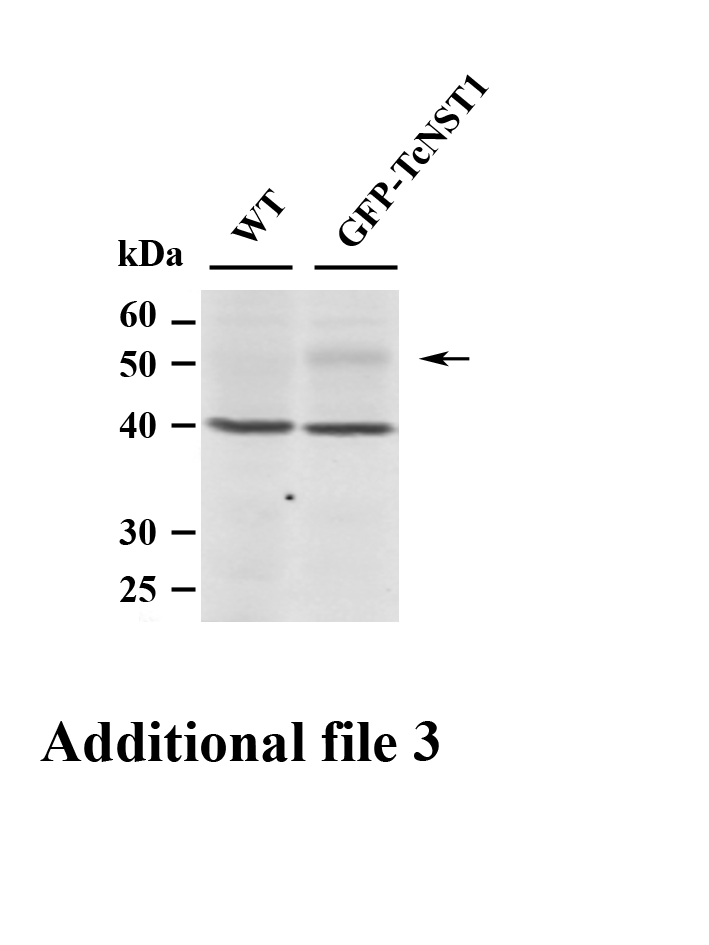

Supplement: Additional file 3: — Expression of GFP-TcNST1 in T. cruzi epimastigotes. Western blot analysis of T. cruzi parasites transfected with GFP-TcNST1 and wild-type cells. Expression of the fusion protein (arrow) was detected with a monoclonal antibody against GFP. Expression of glyceraldehyde 3-phosphate dehydrogenase (GAPDH, ~ 37 kDa) was used as an internal control. (TIF 69 kb) [file 12866_2015_601_MOESM3_ESM.tif]
