# Supplementary material for: Identification of a Golgi-localized UDP-N-acetylglucosamine transporter in Trypanosoma cruzi
Source: BMC Microbiol. 2015 Nov 21;15:269. doi: 10.1186/s12866-015-0601-7 (PMC4654811; doi:10.1186/s12866-015-0601-7)
Supplement: Additional file 4: — Primers used for cloning of the listed genes in different expression vectors. (DOCX 15 kb) [file 12866_2015_601_MOESM4_ESM.docx]

**Table S1**. Primers used for cloning of the listed genes in different expression vectors

| Primer | Gene | Primer sequence (5’ – 3’)^a^ | Vector |
| --- | --- | --- | --- |
| Tc150-F | TcCLB.511517.150 (*TcNST1*) | CGGCCCTCGAGATGAATAAGGAAAGAAATAAG | pE4 |
| Tc150-R2 | TcCLB.511517.150 | CCGGAATTCTTAATGGTGATGGTGATGATGAATCTTTTCTCTTGAATA | pE4 |
| Tc400-F | TcCLB.511277.400 | TCCCCCGGGATGCCAACTTTACAATGGGCT | pE4 |
| Tc400-R2 | TcCLB.511277.400 | CCGGAATTCTCAATGGTGATGGTGATGATGGTGGGTTGGCACGTGAAG | pE4 |
| Tc085.60-F | TcCLB.504085.60 | CGGGCCCTCGAGATGCGGATTTACCTCTTTGGG | pE4 |
| Tc085.60-R | TcCLB.504085.60 | CCGGAATTCTTAATGGTGATGGTGATGATGAGAGTTGTACACAACGATGGC | pE4 |
| Tc120-F | TcCLB.504057.120 | TCCCCCGGGATGAATTCTCTTGGCAATTCG | pE4 |
| Tc120-R | TcCLB.504057.120 | GGCCATCGATCTAATGGTGATGGTGATGATGGATCACGTCCTTTAGTTTTCC | pE4 |
| Tc20-F | TcCLB.509741.20 | TCCCCCGGGATGTCAAAACGCCCATTGGCA | pE4 |
| Tc20-R | TcCLB.509741.20 | CCGGAATTCTTAATGGTGATGGTGATGATGACGCTCCTTACTGCCTTTTTC | pE4 |
| KlUGT-F | Kl UGT | CGGCCCTCGAGATGAGTTTTGTATTGATTTTG | pE4 |
| KlUGT-R | Kl UGT | TCCCCCGGGTCAATGGTGATGGTGATGATGGCGAGGCAGTGCAGT | pE4 |
| Tc150-F | TcCLB.511517.150 | CGGCCCTCGAGATGAATAAGGAAAGAAATAAG | pcDNA 3.1(-) |
| Tc150-R2 | TcCLB.511517.150 | CCGGAATTCTTAATGGTGATGGTGATGATGAATCTTTTCTCTTGAATA | pcDNA 3.1(-) |
| CHO-F | Cg UGT | CTAGTCTAGAATGGCAGCGGTTGGGGTTGGC | pcDNA 3.1(-) |
| CHO-R | Cg UGT | CCGGAATTCCTACGAACCCTTCACCTTGGT | pcDNA 3.1(-) |
| GFP-70F | TcCLB.511517.150 | GGGGACAAGTTTGTACAAAAAAGCAGGCTTCAtgaataaggaaagaaataagtccgggttgtc | pDONR 221 |
| GFP-70R | TcCLB.511517.150 | GGGGACCACTTTGTACAAGAAAGCTGGGTCCTAAATCTTTTCTCTTGAATACGACGGGGCT | pDONR 221 |
| VRFG-F | ScVRG4 | GGGGACAAGTTTGTACAAAAAAGCAGGCTTCATGTCTGAATTGAAAACAGGT | pDONR 221 |
| VRG4-R | ScVRG4 | GGGGACCACTTTGTACAAGAAAGCTGGGTCTATTTACGTAAAGGTTGGGCTTG | pDONR 221 |

^a^ Restriction sites added to the primers and used for cloning are underlined. For the primers Tc400F, Tc120-R and Tc20-F, blunt-end PCR products in the corresponding ends were obtained using Pfx thermopolymerase. The fragments were joined to blunt-ends generated in pE4 after cleavage with either *Xho*I or *Eco*RI and treatment with klenow enzyme. For cloning into the pDONR 221 vector the Gateway Technology (Invitrogen) was used. In this case, underlined sequences correspond to recombination sites.
